# TCDD and Puberty: Warner and Eskenazi Respond

**Published:** 2005-01

**Authors:** Marcella Warner, Brenda Eskenazi

**Affiliations:** School of Public Health, University of California-Berkeley, Berkeley, California, E-mail: mwarner@calmail.berkeley.edu

As Wolff et al. note, in data from the Seveso Women’s Health Study (SWHS) we found no change in age of onset of menarche associated with TCDD exposure in all women in the cohort or in women exposed before 8 years of age (Warner et al. 2004). However, Wolff et al. comment that hormonal exposures before 5 years of age might be the more relevant time period, given that the pubertal transition occurs around 5–7 years of age. Recognizing that our data may be limited by small numbers, Wolff et al. are interested in knowing whether risk of earlier (or later) puberty was seen among girls who were exposed before 5 years of age.

Of the 282 women in the SWHS cohort who were premenarcheal at the time of the explosion on 10 July 1976, 84 women were <5 years of age. The mean age of menarche reported for the 84 women was 12.6 ± 1.5 years, and the median lipid-adjusted serum TCDD level was 233 ppt (range, 3.6–56,000 ppt). In Cox proportional hazards models, when log_10_TCDD was entered as the exposure variable, the hazard ratio associated with a 10-fold increase in TCDD was 1.2 [95% confidence interval, 0.98–1.6; p for trend = 0.07]. That is, the risk of early menarche was increased with the presence of a 10-fold increase in serum TCDD level (e.g., from 10 to 100 ppt), but not significantly. The data were too sparse in the lower exposure groups to perform categorical analyses. The observed increase was limited to the subset of women who were < 5 years of age at exposure, as the effect was diminished when we considered including older ages (< 6 years, < 7 years).

In summary, the sample size is too small to state with certainty, but it seems that the women who received higher exposure and were < 5 years of age at the time of the explosion may have been at somewhat increased risk for earlier menarche. As we stated in our article (Warner et al. 2004), the women in this study experienced significant TCDD exposure during the postnatal but prepubertal developmental period. Given that animal evidence suggests in utero exposure can affect onset of puberty, continued follow-up of the offspring of the SWHS cohort is important.
